# Eukaryotic transcriptomics *in silico*: Optimizing cDNA-AFLP efficiency

**DOI:** 10.1186/1471-2164-10-565

**Published:** 2009-11-30

**Authors:** Kai N Stölting, Gerrit Gort, Christian Wüst, Anthony B Wilson

**Affiliations:** 1 Zoological Museum, University of Zurich, Winterthurerstrasse 190, 8057 Zurich, Switzerland; 2 Wageningen University & Research Center, Bornsesteeg 47, 6708 PD Wageningen, the Netherlands; 3 Institute for Mathematics, University of Zurich, Winterthurerstrasse 190, 8057 Zurich, Switzerland

## Abstract

**Background:**

Complementary-DNA based amplified fragment length polymorphism (cDNA-AFLP) is a commonly used tool for assessing the genetic regulation of traits through the correlation of trait expression with cDNA expression profiles. In spite of the frequent application of this method, studies on the optimization of the cDNA-AFLP assay design are rare and have typically been taxonomically restricted. Here, we model cDNA-AFLPs on all 92 eukaryotic species for which cDNA pools are currently available, using all combinations of eight restriction enzymes standard in cDNA-AFLP screens.

**Results:**

*In silco *simulations reveal that cDNA pool coverage is largely determined by the choice of individual restriction enzymes and that, through the choice of optimal enzyme combinations, coverage can be increased from <40% to 75% without changing the underlying experimental design. We find evidence of phylogenetic signal in the coverage data, which is largely mediated by organismal GC content. There is nonetheless a high degree of consistency in cDNA pool coverage for particular enzyme combinations, indicating that our recommendations should be applicable to most eukaryotic systems. We also explore the relationship between the average observed fragment number per selective AFLP-PCR reaction and the size of the underlying cDNA pool, and show how AFLP experiments can be used to estimate the number of genes expressed in a target tissue.

**Conclusion:**

The insights gained from *in silico *screening of cDNA-AFLPs from a broad sampling of eukaryotes provide a set of guidelines that should help to substantially increase the efficiency of future cDNA-AFLP experiments in eukaryotes. *In silico *simulations also suggest a novel use of cDNA-AFLP screens to determine the number of transcripts expressed in a target tissue, an application that should be invaluable as next-generation sequencing technologies are adapted for differential display.

## Background

Researchers interested in studying the genetic regulation of particular processes or traits must first identify the genes contributing to the phenotype, a step which can be particularly challenging in organisms for which genomic data are not yet available. Differential display methods have been commonly used to compare levels of gene expression in target tissues at various stages, allowing the identification of sets of genes whose expression patterns are significantly correlated with traits of interest [1].

Among the available differential display methods, one increasingly popular tool is cDNA-amplified fragment length polymorphism (cDNA-AFLP, [2]). This method allows the identification of differences in the expression of genes that are correlated to a trait of interest and has proven particularly useful in non-model organisms, as it does not require previous sequence knowledge. The cDNA-AFLP technique involves the digestion of cDNA preparations produced from RNA extractions with two restriction enzymes. To analyze the produced fragments, adaptors are ligated to each restriction fragment, which then serve as oligonucleotide-binding sites for two subsequent rounds of PCR. By adding a few (typically <4), selective base pairs (bp) to these primer sequences, the amplified fragment pool is reduced in complexity such that a suitable number of fragments can be visualized [3,4]. By comparing the presence or absence of individual fragments in individual cDNA libraries after size separation, one can identify genes correlated to the trait of interest. While the use of traditional gels (agarose, acrylamide, spreadex, etc.) is required for the recovery of fragments for further characterization, separation on fluorescent sequencers allows for high throughput and has become standard [4].

A well-designed differential display experiment should aim to sample all transcripts present in a target tissue in order to avoid biasing downstream analyses. Optimizing coverage (here defined as the fraction of sequences that appear at least once as fragments of resolvable size (50-500 bp) in an exhaustive cDNA-AFLP screen) is at the heart of designing a successful experiment. Insufficient coverage of the cDNA pool can prevent the detection of genes correlated to the trait of interest, even if gene expression differences underlie trait production. Although complete pool coverage may often not be possible in any differential display screen, the recent literature indicates that dozens to hundreds of transcript-derived fragments (TDF) correlated to traits of interest can be obtained from the successful application of cDNA-AFLP screens (Table 1). A variety of modified cDNA-AFLP protocols have been proposed to optimize pool screening [5-7].

**Table 1:** Results of cDNA-AFLP screens from ten recent publications.

RE1	RE2	PC	TDFs	Mean TDF	Corr. TDF	Reference
BstYI	MseI	60	4000	66.67	63	[29]
BstYI	MseI	64	3793	59.27	213	[30]
BstYI	MseI	128	10440	81.56	223	[31]
BstYI	MseI	128	7000	54.69	1196	[32]
BstYI	MseI	256	5900	23.05	378	[33]
EcoRI	MseI	64	3220	50.31	34	[34]
EcoRI	MseI	128	2269	17.73	25	[35]
EcoRI	MseI	256	12500	48.83	525	[36]
HindIII	MseI	32	4320	135.00	26	[37]
PstI	MseI	80	1200	15.00	46	[38]

	***Average***	119.6	5464.2	55.2	272.9	
	***Median***	104.0	4160.0	52.5	138.0	

TDFs are the number of transcript-derived fragments produced in each screen, while PC indicates the number of primer combinations tested in each study. Mean TDF indicates the average numbers of fragments generated per primer combination, while Corr TDF identifies the number of transcript-derived fragments that were found to be correlated to the trait under investigation. Restriction enzymes (RE) listed in column RE1 are characterized by recognition sites of 6 bp, while RE2 (here: MseI) is a 4 bp-cutter.

The absolute number of TDFs that are screened per selective amplification is determined by several factors. An increase in the number of selective base pairs will reduce the number of fragments screened per PCR, and the choice of appropriate restriction enzymes can also systematically and substantially affect the quality of a screen, due to functional or evolutionary constraints on the triplets of amino-acid coding cDNA. The total number of fragments obtained is also directly linked to the total cDNA pool size, because the presence of more (different) cDNAs provides more restriction sites, and thus a larger pool is expected to produce more fragments per PCR. It is intuitively appealing to simply maximize the number of fragments screened per PCR to minimize the workload, and in fact the first AFLP studies [3] suggested that up to 100 AFLP fragments could be reliably separated. However, subsequent studies have shown that when the number of fragments visualized exceeds ~20 per PCR, there is a significant risk of co-migrating fragments that can confound the reliability of an AFLP screen [8]. The risk of co-migration is further complicated by the fact that sequences of different lengths may migrate together for a variety of reasons, including physical damage to the DNA molecule, differences in base pair composition and/or methylation [9]. For all of these reasons, studies in which the accuracy of AFLP-scoring is critical need to be particularly sensitive to the risks of high-throughput analysis.

Complementary DNA-AFLP optimization problems can be addressed by computational (*in silico*) analysis. These *in silico *approaches are becoming increasingly feasible as genetic databases increase in taxonomic breadth, analytical tools are developed, and computational resources increase in power. As AFLP searches are essentially searches for particular sequence motifs, the implementation of cDNA-AFLP *in silico *is computationally straightforward. Each of these screened sequence motifs is composed of the recognition site of the restriction enzyme and three or fewer selective base pairs, such that analyses are restricted to searches for up to 4^3 ^× 4^3 ^= 4096 sequence motifs for a three-selective base pair experiment involving two enzymes.

The first quantitative cDNA-AFLP *in silico *studies approached this optimization problem in individual taxa, identifying several factors that can improve experimental design. Kivioja et al. [[6], Kivioja, unpublished data] suggested that the use of restriction enzymes with 6-bp restriction sites is likely to be disadvantageous in cDNA-AFLP studies due to the fact that such enzymes significantly reduce pool coverage. Again, simply maximizing the number of fragments screened per selective PCR by using restriction enzymes that cut frequently is not necessarily optimal, as this increases the risk of obtaining size-homoplasious fragments (henceforth: collisions) within each selective amplification [8]. There is thus a tradeoff between data quantity and quality in cDNA-AFLP experiments. Methods have been proposed which would minimize the number of amplifications required per enzyme combination [6] when the cDNA pool has been previously characterized, but it is unclear whether these approaches have more widespread applicability.

These first *in silico *approaches to the study of cDNA-AFLPs suffer from two significant limitations. First, these studies used cDNA data from a small number of (often closely related) taxa [5-8], an approach that could restrict the wider applicability of their conclusions, as codon usage is known to vary widely across taxonomic groups [10]. As one of the major benefits of AFLPs is their ready applicability to new taxa, this may be a particularly important issue. A second potential limitation of these earlier studies stems from the fact that previous *in silico *analyses of cDNA-AFLPs used RefSeq sequences from curated resources, which are typically biased towards larger and more complete sequences. As this quality of data is rarely available in real-world datasets, insights gained from simulations based on these data may not be relevant for typical research projects. The effects of the raw data themselves on the outcome of the *in silico *optimizations have not yet been unexplored.

To overcome the limitations of previous *in silico *studies, we use a taxonomically diverse eukaryotic dataset to investigate traditional cDNA-AFLP experiments *sensu *Bachem [11]. Briefly, cDNA is digested with two restriction enzymes, from which subsets of fragments are amplified and then separated by electrophoresis. Depending on the frequency of restriction enzyme cleavage, multiple fragments may be generated for each cDNA. We maximize cDNA pool coverage and optimize the number of TDFs produced per selective PCR using simulated cDNA-AFLPs on a wide taxonomic sampling of 92 eukaryotic species representing most major groups (See additional file 1: "General information for each species" and additional file 2 "Species composition of included taxonomic groups"). Making use of data from two different repositories, we also investigate whether systematic differences exist between datasets obtained from different databases. After quantifying these effects, we test all 28 combinations of eight commonly used restriction enzymes on all 92 species and assess the relative performance of individual enzymes on cDNA-AFLP screens. By including information on the taxonomic grouping of each species, we are able to investigate whether there is significant phylogenetic signal in the data, a finding which could indicate that different cDNA-AFLP protocols might be necessary for particular taxonomic groups. This quantitative dataset is then used to compare and identify optimal enzyme combinations, both at the species-level and across all eukaryotes.

The cDNA pool coverages obtained in these global analyses are based on the execution of all possible selective PCRs, but such extensive screens are often infeasible in the laboratory. To investigate potential differences in TDF recovery during selective PCR, we simulate all possible combinations of selective PCRs for each enzyme combination and species and extract information on the number of fragments produced per selective PCR. Because the maximum number of selective amplifications is frequently limited and the selective base pairs used in amplifications are not necessarily independent of each other, we use graphical representations to identify general patterns in the performance of selective amplifications. As a comparison, we perform *in silico *AFLP on simulated DNA and cDNA datasets to address whether cDNA-AFLP patterning in real data differs from neutral expectations.

Our comprehensive *in silico *approach provides a realistic quantitative framework for the design of future cDNA-AFLP experiments. In addition to removing the guesswork from the design of such screens for non-model organisms, our *in silico *approach offers a powerful means for identifying general patterns in the transcriptomes of both model- and non-model species.

## Results

### Consistent results from curated datasets

NCBI and ENSEMBL databases provided a total of 113 pools of cDNA for this study. Twenty-one species were present in both databases, providing an opportunity to investigate the potential effects of database origin on pool coverage. While the data from NCBI and ENSEMBL differed significantly in many characteristics (See additional file 3: "Duplicate species from ENSEMBL and NCBI databases"), the source of the data did not explain a significant proportion of the variation in cDNA pool coverage after controlling for total pool size, average sequence length, GC content and the proportion of ambiguous nucleotides (See additional file 3 and additional file 4: "Influence of database origin on pool coverage"). Duplicated species from the NCBI database were therefore removed to avoid pseudo-replication in subsequent analyses (see Methods).

### Sources of variability in cDNA pool coverage

Considerable variability exists in the observed cDNA pool coverage both within and across species (Table 2; see also additional files 1 and 3). Two major sources of variability in coverage can be identified. Sequence characteristics such as average cDNA length and the total pool size explain a significant proportion of the variation in the pool coverage. Of these technical effects, average sequence length explains 38% of the variation in cDNA pool coverage. Less important is the effect of total pool size (14.3% of the variation in coverage explained), while the effect of ambiguous bases on pool coverage is non-significant (Table 2).

**Table 2:** The relative contribution of enzyme combinations to cDNA pool coverage.

Source	Num df	Den df	F	Sig.	**Partial R**^**2**^
Model	58	2248.56	134.76	<0.001	77.66
Total pool size (bp)	1	85.40	14.25	<0.001	14.30
Average sequence length	1	86.29	52.87	<0.001	37.99
GC content	1	86.60	34.89	<0.001	28.72
Non-ACGT content	1	84.49	0.05	0.823	<0.01
Enzyme combination	27	2428.48	199.26	<0.001	68.90
Enzyme combination*GC content	27	2428.48	112.38	<0.001	55.55

Variance partitioning addressing the contribution of enzyme combination (28 combinations) on pool coverage for 92 eukaryotic species (see additional file 1). Species was included as a random factor in a mixed model analysis which aimed to determine the influence of individual factors or interactions (Source). cDNA pool coverage was weighted by the number of sequences per species to account for variation in available sequence data. The numerator and denominator (Kenward-Roger corrected) degrees of freedom (Num df/Den df) are provided. F statistics (F) and the significance (Sig.) of the overall model, factors and interactions are reported. The proportion of the variation in cDNA pool coverage which is explained by each factor/interaction is indicated as Partial R-square values [27].

A larger portion of the variation in coverage can be explained by biological factors (Table 2), of which the combination of restriction enzymes is most important, explaining 68.9% of the observed variation in coverage. The GC content of the target species explains 28.7% of cDNA pool coverage, and a significant two-way interaction exists between enzyme combination and the GC content of the pool, explaining 55.6% of the variation in coverage. This significant interaction term indicates that optimal enzyme combinations differ among species (see also additional file 1) and suggests that GC content should be considered when choosing optimal restriction enzymes for a cDNA-AFLP screen. Taken together, our mixed model explains 78% of variation in cDNA pool coverage (Table 2).

The choice of the most appropriate restriction enzymes substantially increases the coverage of a given cDNA pool from less than 40% to more than 75% (Table 3). The effects of restriction enzymes are essentially additive (compare Table 3 and Table 4), indicating that the performance of individual restriction enzymes is not strongly influenced by the second enzyme used in the double digest.

**Table 3:** Average cDNA pool coverages by enzyme combination across 92 eukaryotes.

Enzyme Combination	Coverage ± SD	Min-Max Coverage	**R**^**2**^	Regression Equation
MseI & CviAII	76.13 ± 10.51	42.07 - 91.97	0.94	Nbp = 1849399*AF+294835
CviAII & CviQI	72.55 ± 10.32	46.44 - 93.68	0.98	Nbp = 1924913*AF+1067365
CviAII & TaqI	69.56 ± 13.62	32.19 - 96.94	0.85	Nbp = 2152800*AF+933639
MseI & CviQI	66.63 ± 10.32	36.63 - 86.15	0.94	Nbp = 2731950*AF-862614
CviAII & MaeII	64.16 ± 13.93	33.34 - 91.88	0.91	Nbp = 2309466*AF+1759142
MaeI & CviAII	63.20 ± 11.42	21.81 - 85.13	0.90	Nbp = 2306663*AF+3307692
MseI & TaqI	62.69 ± 14.23	28.35 - 90.91	0.72	Nbp = 2995858*AF+626737
HpaII & CviAII	62.00 ± 18.32	9.45 - 93.30	0.94	Nbp = 1890564*AF+3666024
TaqI & CviQI	61.26 ± 14.41	25.91 - 94.55	0.76	Nbp = 2726475*AF+2079328
MseI & MaeI	58.28 ± 12.22	27.61 - 79.49	0.84	Nbp = 2852998*AF+2622539
MseI & MaeII	57.15 ± 12.86	29.81 - 84.18	0.85	Nbp = 3630319*AF-565133
MaeII & CviQI	55.70 ± 15.43	23.57 - 88.18	0.85	Nbp = 3156237*AF+1906975
MaeI & CviQI	54.91 ± 11.08	21.14 - 81.68	0.91	Nbp = 3400108*AF+2063017
TaqI & MaeII	54.86 ± 17.23	19.16 - 92.44	0.64	Nbp = 2762584*AF+4754785
HpaII & MseI	54.39 ± 14.73	12.13 - 87.81	0.92	Nbp = 3607697*AF+484514
HpaII & CviQI	54.14 ± 18.34	9.05 - 91.74	0.88	Nbp = 2623528*AF+3657451
HinP1I & CviAII	53.57 ± 19.96	5.08 - 95.05	0.85	Nbp = 2260683*AF+4996193
HpaII & TaqI	53.54 ± 19.47	8.04 - 93.28	0.72	Nbp = 2433331*AF+5040020
MaeI & TaqI	52.26 ± 13.47	17.71 - 85.30	0.80	Nbp = 4183276*AF+518528
HpaII & MaeII	49.93 ± 19.70	5.49 - 89.29	0.79	Nbp = 2690086*AF+4940474
HinP1I & CviQI	47.88 ± 19.79	5.27 - 89.77	0.79	Nbp = 2909507*AF+5271739
MaeI & MaeII	47.76 ± 12.04	15.32 - 78.81	0.93	Nbp = 4923589*AF-278386
HinP1I & TaqI	47.32 ± 20.70	4.26 - 91.43	0.63	Nbp = 2600056*AF+7269281
HinP1I & MseI	46.22 ± 15.07	6.86 - 87.60	0.84	Nbp = 4211767*AF+2594577
HpaII & MaeI	45.75 ± 15.11	4.16 - 80.64	0.92	Nbp = 4048888*AF+3106105
HinP1I & HpaII	45.05 ± 22.78	1.36 - 94.75	0.78	Nbp = 2272295*AF+6925901
HinP1I & MaeII	44.15 ± 20.69	4.08 - 89.75	0.71	Nbp = 2937393*AF+6703632
HinP1I & MaeI	39.00 ± 15.30	2.53 - 77.47	0.89	Nbp = 5159080*AF+3260514

Descriptive statistics on the average cDNA pool coverage obtained for each enzyme combination across all 92 species (see additional file 1), sorted by decreasing mean coverage. The average coverage by enzyme combination and standard deviation (Coverage ± SD) are indicated, as are the minimum and maximum cDNA pool coverages for individual enzyme combinations (Min-Max Coverage). R-Square indicates the correlation coefficient for the relationship of total cDNA pool size (Nbp) and the average number of fragments produced per selective PCR (AF). The linear regression equation for this relationship is indicated.

**Table 4:** Effects of individual restriction enzymes on cDNA-pool coverage.

Source (Restriction Site)	df	SS I	F	Sig.	Coverage Estimate
CviAII (C^ATG)	1	121.08	4806.32	<0.001	43.63
MseI (T^TAA)	1	153.42	6090.19	<0.001	32.49
CviQI (G^TAC)	1	69.09	2742.53	<0.001	31.06
TaqI (T^CGA)	1	52.69	2091.52	<0.001	30.20
MaeI (C^TAG)	1	63.15	2506.75	<0.001	25.79
MaeII (A^CGT)	1	86.56	3436.05	<0.001	24.66
HpaII (C^CGG)	1	136.20	5406.74	<0.001	23.34
HinP1I (G^CGC)	1	137.28	5449.32	<0.001	17.12
Enzyme combination	20	0.78	1.55	0.056	n/a

The effects of individual restriction enzymes on cDNA-pool coverage, based on all 92 species (Table 3; see additional file 1). The percentage of total cDNA pool coverage explained by each enzyme has been estimated. The degrees of freedom (df) of each factor included in the model (source) are indicated. Enzymes are sorted by decreasing coverage, and restriction sites of each restriction enzyme are listed. Details on the significance of each factor (Sig.) in this analysis and corresponding F-statistics are given (see Methods).

### Effects of evolutionary history on cDNA-pool coverage

Analyzing sequence data from a group of organisms with an evolutionary history as old and diverse as that of eukaryotes allows the quantification of the effects of taxonomic substructure on cDNA pool coverage. 68 of the 92 study species could be assigned to eight major taxonomic groups (see also additional file 2) with at least three members per group. This additional predictor (taxonomic group) improves the fit of our model by 16.1% (Table 5). Taxonomic grouping itself explains 62.2% of the variation in pool coverage. Once again, the choice of enzyme combination explains the highest proportion of coverage in this model (79.6%), and the influence of technical effects is less significant. Of these sequence characteristics, the average sequence length has again the strongest influence and explains 54.0% of the variation in cDNA pool coverage, while the total pool size accounts for only 8.2% and the proportion of ambiguous nucleotides does not significantly affect coverage. There is a strong interaction between taxonomic group and enzyme combination (p < 0.001) indicating that the optimal enzyme combination varies across groups (see also additional file 2). This difference is mediated in large part by differences in GC content among the taxa included here (70.7% variation explained; Table 5).

**Table 5:** Effects of taxonomic grouping and enzyme combination on pool coverage.

Source	Num df	Den df	F	Sig.	**Partial R**^**2**^
Model	254	1485.02	87.57	<0.001	93.74
Taxonomic group	7	57.37	13.48	<0.001	62.19
Total pool size (bp)	1	55.88	5.00	0.029	8.22
Average sequence length	1	56.31	66.16	<0.001	54.02
GC content	1	56.56	12.12	0.001	17.65
Non-ACGT content	1	56.59	0.75	0.389	1.31
Enzyme combination	27	1593.69	230.17	<0.001	79.59
Enzyme combination * GC content	27	1593.69	142.71	<0.001	70.74
Enzyme combination * Taxonomic group	189	1593.69	21.05	<0.001	71.40

Variance partitioning addressing the influence of enzyme combination (28 combinations) and taxonomic grouping on pool coverage for 68 species (see additional file 2). Species was included as a random factor and cDNA pool coverage was weighted by the number of sequences per species to account for variation in available sequence data. Denominator degrees of freedom were Kenward-Roger corrected. Partial R-square indicates the proportion of the variation in cDNA pool coverage which is explained by each factor/interaction [27].

### A positive relationship between cDNA-AFLP fragment number and pool size

**Figure 1:**
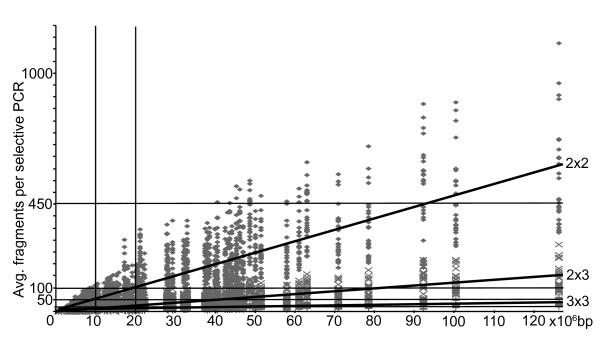
**A positive relationship between cDNA pool size and the number of fragments per PCR**. Linear regressions of average fragment numbers produced during *in silico *selective cDNA-AFLP PCRs against the absolute cDNA pool size in bp. Symbols indicate the average fragment numbers produced per enzyme combination and species for selective amplifications using 2 × 2 (diamonds), 2 × 3 (crosses) and 3 × 3 (pluses) selective base pairs, respectively. Duplicate species have been removed from this analysis. The numbers of selective base pairs used for each primer in the selective PCR are indicated, and regression lines have been added for each of the three amplification types. The correlation coefficient for each of the three datasets is 0.74. The production of fewer than 20 fragments per PCR minimizes the possibility of collisions [8], while up to 100 fragments per reaction are often desired when performing AFLP on genomic DNA [3]. A maximum of 450 fragments can be separated in the typical size range of AFLP screens (50-500 bp). Vertical reference lines indicate the total cDNA pool size range expected in a typical tissue expressing between 7500 and 15000 different cDNAs [24] assuming an average cDNA length of 1346 bp [12].

We were interested to see whether a relationship exists between the average number of fragments produced per selective PCR and any of the additional information we collected for each cDNA pool. We found a strong positive correlation between the average fragment number per selective PCR and the size of the cDNA pool in base pairs (Figure 1, Table 3). With an r^2 ^of 0.63 - 0.98, the average fragment counts generated per PCR provide a reasonable estimate of the size of the underlying cDNA pool.

cDNA length averaged 1113 ± 489 bp across the pools included in the present study (see additional file 1), similar to the recently published estimate of 1346 bp derived from gene predictions in the eukaryotic genome [12]. Using these estimates, it is possible to convert the estimated total pool sizes in base pairs into absolute numbers of cDNAs. The linear relationship between total cDNA pool size and average fragment number per selective PCR can help minimize the possibility of collisions when optimizing cDNA-AFLP experimental design. In case of a selective PCR regime which employs a two-by-three selective base pair design, the threshold of 20 fragments per PCR reaction to minimize the chance of collisions will rarely be reached in tissues with fewer than 15000 sequences, assuming an average cDNA length of 1346 bp. However, the frequently used two-by-two selective base pair design will yield more than 20 fragments per selective PCR in a pool of only 7500 cDNAs and nearly 100 fragments in a pool of 15000 cDNAs (Figure 1), suggesting that a two-by-two selective base pair design is likely to introduce a significant source of error via collisions in a typical cDNA screen [8].

### Non-random patterning in cDNA-AFLP arrays

**Figure 2:**
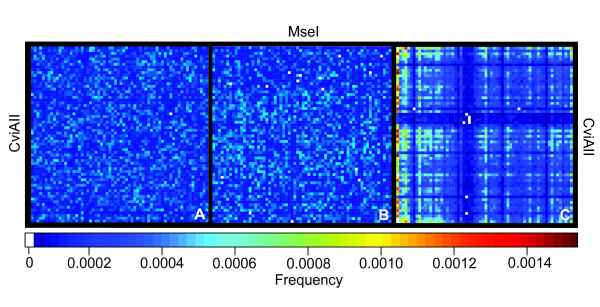
**Empirical cDNA-AFLP data are highly structured**. Patterning of cDNA-AFLP data. A and B: Patterning of complete arrays of selective PCR amplifications using CviAII and MseI restriction enzymes for (A) simulated random DNA, (B) simulated cDNA (following the standard eukaryotic codon table [25]), and (C) *Homo sapiens *cDNA. 10000 sequences of 1290 bp were simulated for both the DNA and cDNA datasets. Pixel intensity reflects the relative proportion of products obtained during selective *in silico *PCR. Pixels are ordered by selective base pairs: AAA (left, top) to TTT (bottom, right). White pixels indicate that no fragments were generated for this combination of selective base pairs.

**Figure 3:**
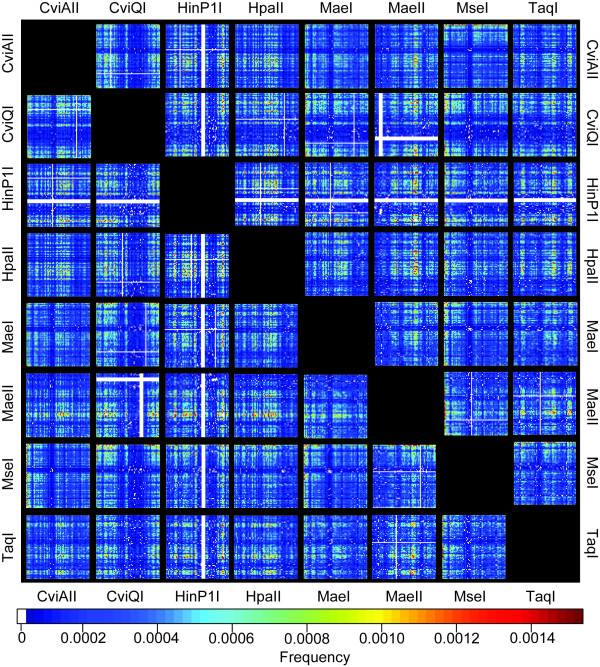
**Characteristic cDNA-AFLP patterns are generated by individual restriction enzymes**. Overview of the *Homo sapiens *selective cDNA-AFLP PCR arrays for all enzyme combinations tested here. The layout of arrays follows Figure 2. Note the consistent patterning of arrays, with characteristic ridges and trenches for enzyme combinations which contain the same enzyme. Arrays above the diagonal are mirror images of those below the diagonal. Selective primer combinations yielding no amplifications are highlighted in white. The pixel intensity indicates the relative proportion of fragments amplified in a given selective PCR combination.

**Figure 4:**
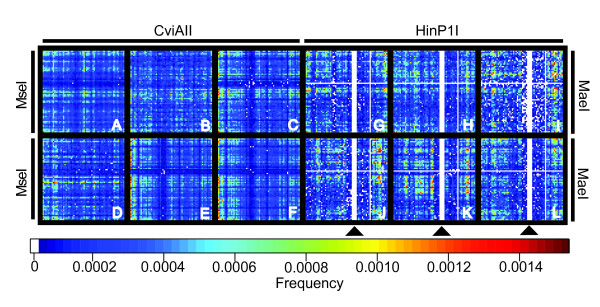
**cDNA-AFLP patterning is consistent across all eukaryotes**. Arrays of all possible cDNA-AFLP selective PCR combinations for the best (A-F) and worst (G-L) restriction enzyme combinations. Six species per enzyme combination are included. A-F restriction enzymes CviAII and MseI, G-L restriction enzymes HinP1I and MaeI. A/G *Arabidopsis thaliana*, B/H *Drosophila melanogaster*, C/I *Gallus gallus*, D/J *Gasterosteus aculeatus*, E/K *Homo sapiens*, F/L *Xenopus laevis*. Arrowheads pointing to white areas in the arrays indicate primer combinations with GCN-selective base pair motifs, which fail to produce any fragments in a cDNA-AFLP screen with these enzymes (see Discussion).

Selective PCRs generally use up to three selective base pairs, and hence a maximum of 4^3 ^× 4^3 ^= 4096 different selective amplifications are theoretically possible when using two restriction enzymes. According to neutral expectations, each of these selective primer combinations would be expected to produce on average a similar number of fragments. We used array plotting to visualize the relative fragment numbers produced by each potential selective PCR in the typical three-by-three selective base pair design and found considerable structure in empirical data that is not found in simulated cDNA and genomic DNA pools (Figure 2). Such structure is observable for all enzyme combinations (e.g. *Homo sapiens*; Figure 3). As is apparent from Figure 3, restriction-enzyme specific patterning for individual enzymes is highly conserved even when enzymes are used in different combinations, suggesting that the difference between the fragment numbers per selective PCR is largely the result of the individual restriction enzymes (see above). Particular selective PCRs fail to generate any products and are thus entirely uninformative in cDNA-AFLP screens. In these cases, one or both restriction enzymes cut closely together, producing AFLP products too small to be visualized in the screen (see Discussion). This restriction-enzyme patterning is consistent even in distantly related taxa (Figure 4), indicating the strong signal of evolutionary history in the underlying datasets.

## Discussion

Complementary DNA-AFLPs are an increasingly popular tool to study differential gene expression, particularly in non-model organisms for which genome data are unavailable (Table 1). The main benefits of the cDNA-AFLP approach are the relative ease of its implementation and its low per-marker costs [13]. In addition to the traditional use of cDNA-AFLPs to identify dominant (i.e. presence-absence) markers correlating to traits of interest, recent methods have shown that cDNA-AFLPs can also provide quantitative data [14]. Regardless of the goals of a cDNA-AFLP experiment, a successful screen requires high coverage of the underlying cDNA pool. While significant advances have been made in technical aspects of the AFLP methodology, theoretical studies investigating methods for optimizing the cDNA-AFLP screens remain relatively rare, and large scale empirical data - as provided here for eukaryotes - have not yet been used for this purpose [6-8].

Recent years have seen an explosion in cDNA datasets. ENSEMBL and NCBI are two of the most important repositories for cDNA data, and the taxonomic coverage and quality of data in these archives will continue to grow with the development of next-generation sequencing technologies. Given the vast amount of available data - in the present study a total of more than 1.7 million sequences and 2.2 Gbp of cDNA were screened - *in silico *studies offer the potential to address novel research questions and to optimize experimental protocols before undertaking large experimental studies. The cDNA pools included in the present study cover most major extant eukaryotic groups, providing an opportunity to identify broadly applicable conclusions on the most important factors affecting the quality of cDNA-AFLP screens. These cDNA pools range from a few hundred to more than 57,000 sequences (see additional file 1), covering the range of experiments likely to be undertaken in both model- and non-model organisms.

Using previously published and pre-filtered data has the potential to introduce technical artifacts into *in silico *analyses. The database origin of cDNA pools does not affect our coverage optimization after controlling for differences in sequence length, total pool size, GC content and the proportion of ambiguous nucleotides (see additional file 4: "Influence of database origin on pool coverage"). When comparing data derived from different databases, non-ACGT content was found to explain a significant component of pool coverage (see additional file 4). This result is due to an abnormally high proportion of ambiguous nucleotides in the *Gasterosteus aculateus *cDNA pool obtained from the NCBI repository (1.26%, versus 6 × 10^-6^% in the ENSEMBL dataset; see also additional file 3). This effect of non-ACGT nucleotides on coverage disappears when this species is removed from the analysis (data not shown).

cDNA pool coverage in the complete dataset of 92 species (see additional file 1) is significantly affected by both total pool size and average sequence length, which explain 14% and 38% percent of coverage, respectively (Table 2). Because the cDNA-AFLP method requires the presence of at least two restriction sites in proximity to screen each transcript, cDNA sequence length can have a large effect, and a significant reduction in coverage is expected when using short cDNA sequences. While the quality of the cDNA preparation can influence cDNA length, differences in cDNA length between species may also reflect biological reality. Species included in our study differ substantially in average cDNA sequence length (see additional file 1). This difference is most pronounced between plants (coniferopsids, liliopsids and streptophytes), which have an average cDNA length of approximately 800 bp, and mammals, which have an average cDNA sequence length of 1600 bp (see additional file 2). This difference, though more modest, is also evident in the results of recent full-length cDNA sequencing projects. An average cDNA length of ~1.5 kb has been reported in plants [e.g. [15-18]], whereas mammals have on average longer full length cDNAs of ~1.7 kb [e.g. [19-23]]. While these studies indicate cDNA length may vary among taxonomic groups, the biological implications and evolutionary consequences of this variation remain unclear.

Technical issues have an important effect on the outcome of cDNA-AFLP experiments, but the restriction enzymes employed explain the majority of the variation in pool coverage (Table 2, Table 5). Here, three factors are relevant. First, the use of restriction enzymes with 6-bp recognition sites is not recommended for cDNA pools [[6], Kivioja, unpublished data], as it greatly reduces the number of fragments generated per PCR reaction. Second, among the restriction enzymes tested here, some are far better suited for cDNA-AFLPs than are others. Estimates of the effects of individual enzymes on coverage (Table 4) or their combined effect (Table 3) clearly indicate that the efficiency of the pool coverage can be nearly doubled by choosing the optimal enzyme combination. Of the restriction enzymes included here, CviAII, MseI and CviQI outperform the other enzymes and are as such good candidates for cDNA-AFLP screens in eukaryotes (Table 3, Table 4). Finally, several basic rules should be kept in mind when choosing restriction enzymes. A strong interaction between optimal restriction enzymes and organismal GC content is apparent in all analyses (see also additional file 2). Clearly, restriction enzymes with GC-rich recognition sites are likely to cut more frequently in GC rich genomes than in those with reduced GC content. Similarly, the use of restriction enzymes with recognition sites frequently found in cDNAs could likewise aid in obtaining in-depth pool coverage. As most previous studies have used a six-cutter restriction enzyme together with a four-cutter and have focused on a small number of primer combinations (Table 1), the number of genes correlated to traits of interest has likely been frequently underestimated.

Complementary DNA-AFLPs have been applied to a wide range of eukaryotic taxa, and the ease of implementing this method in new systems is one of its particular strengths. While previous studies proposed suitable enzyme combinations for species for which sequence data are already available [6], the restricted taxonomic focus of these earlier studies limited the applicability of inferences across a wider array of organisms. As can be seen from Table 5, significant effects of taxonomic grouping exist, and a strong interaction between the taxonomic grouping and the GC content is apparent (compare Table 2 with Table 5). While this indicates that the optimal choice of restriction enzymes differs among taxonomic groups, it also indicates that a large portion of this difference in optimal enzyme choice can be explained by organismal GC content (see additional file 2). By considering GC content prior to undertaking a cDNA-AFLP experiment, researchers should be able to optimize the quality of their screens.

Our *in silico *experiment revealed that cDNA-AFLP performance differs markedly from neutral expectations (Figure 2) and that the observed patterning is highly consistent across taxa (Figure 4). Clearly, cDNA pool coverage could be even further enhanced through a more explicit incorporation of the results presented here. By selecting only the best performing selective base pair combinations for several independent enzyme pairs, one should be able to maximize pool coverage in a reasonably-sized cDNA-AFLP experiment. We refer the reader to additional file 5: "Arrays of all selective PCRs for all species and enzyme combinations", which provides complete cDNA-AFLP arrays for all species investigated here. Figure 3 indicates that most of this patterning results from the effects of the individual restriction enzymes. This is especially apparent for areas of uninformative selective primer combinations in which particular primer-enzyme combinations fail to generate any cDNA-AFLP products at all. This pattern is a result of the AFLP methodology, where restriction enzymes are used to digest double-stranded DNA and adaptors are ligated directly to the digested cDNA ends. During selective amplifications, the selective base pairs of each primer extend directly 3' from the recognition site. As a consequence, an AFLP screen using four-cutter enzymes and three selective base pairs is equivalent to a motif search for DNA stretches of 7-bp length. When restriction enzymes overlap in one or more base pairs, this motif may contain multiple restriction enzyme recognition sites, producing cDNA fragments shorter than the 50 bp required for visualization. These classes of selective PCRs will thus not produce any fragments of mixed type. The selective amplification of HinP1I-generated fragments with the selective base pairs GCN is one such example (Figure 4). When a given DNA sequence contains the motif GCGCGCN, HinP1I will cleave the sequence at two positions (G^CGC^GCN). Due to this double digest, the use of HinP1I will fail to generate any AFLP fragments containing the GCGCGCN motif. Even when this overlap in recognition sites is only partial, the number of fragments generated by a particular pair of selective primers can be reduced, which might explain a portion of the observed patterning. However, the absence of patterning in the simulated data relative to *Homo sapiens *(Figure 2) suggests that technical aspects of the cDNA-AFLP method are insufficient to explain the higher level of complexity found in real data. As this structure is remarkably consistent across taxa (Figure 4), factors highly conserved across evolution (such as codon usage) must contribute to this pattern.

During AFLP screens, selective PCRs are used to reduce the complexity of produced fragment pools. The average number of fragments produced during each selective PCR is positively correlated with the size of the cDNA pool (Figure 1, Table 3). For the restriction enzyme combinations investigated here, the average number of fragments obtained from selective PCRs can be converted into an estimate of the - typically unknown - size of the underlying cDNA pool. This novel versatility of the AFLP methodology - estimating cDNA pool size - should be particularly useful for any study in which knowledge of the underlying transcriptome size is critical. This is especially the case when performing large scale sequencing of the transcriptome, where a preliminary cDNA-AFLP screen may offer a cost-effective means to estimate the number of genes expressed in a tissue of interest.

The linear relationship between average fragment number and total cDNA pool size can also provide guidance when deciding on how many selective base pairs to use. From Figure 1 it is apparent that a two-by-two selective base pair design will often result in fragment numbers that far exceed that optimal for reliable fragment separation (<100 fragments per amplification) or to avoid significant homoplasy (<20 fragments per PCR). A three-by-three selective base pair design is, however, too conservative, in that too few fragments will be screened per PCR reaction (less than 10 fragments per PCR will be generated for datasets containing the equivalent of up to 15000 cDNAs - about 20 Mbp of cDNA sequence). Using a two-by-three selective base pair design appears to be the best option for most cDNA screens, producing 10-20 fragments per amplification (Figure 1; [8]) in cDNA pools of up to 15000 sequences or 20 Mbp, pool sizes expected *in vitro *in typical mammalian tissues [24].

## Conclusion

### Optimizing the quality of cDNA-AFLP screens

Our *in silico *approach to cDNA-AFLP optimization suggests several key improvements to existing methods of cDNA-AFLP experiments and highlights restriction enzymes likely to be particularly well suited for screening eukaryotes (Table 4, see additional file 1). Matching the GC content of the restriction enzymes with that of the target cDNA is a relatively simple step to optimize experimental design. Consideration of the restriction enzyme recognition sites is particularly important, especially when resources limit the number of selective PCRs that can be performed. Following these recommendations will significantly improve the efficiency of future cDNA-AFLP experiments.

### A new application of the cDNA-AFLP methodology

In addition to our methodological suggestions, the comparative approach taken here identified a positive linear relationship between the average fragment numbers per selective PCR and the size of the underlying cDNA pool. This provides a novel method to estimate the number of transcripts present in a cDNA pool via a simple series of cDNA-AFLP screens, an application which will be invaluable as next generation sequencing technologies are adapted for differential display.

## Methods

### Sampling scheme

An *in silico *routine for AFLPs [5] was modified here to simulate the AFLP procedure on cDNA datasets. We included the 39 eukaryotic species available from the ENSEMBL repository http://www.ensembl.org/info/data/ftp/index.html as well as all 87 NCBIftp://ftp.ncbi.nih.gov/repository/UniGene/ cDNA datasets available as of January 2008, providing a taxonomic sample covering all available eukaryotic species. We chose these databases because the frequently used RefSeq databases [6,7] lack alternative splice variants, incomplete genes and pseudogenes, sources of cDNA variation commonly present in real world data. As such, our *in silico *optimization of the cDNA-AFLP routine is a much more realistic approximation of experimental (*in vitro*) conditions. As we wish to help the experimenter in designing experiments for their own target species, our data are based on whole organism cDNA equivalents rather than tissue-specific datasets, for which available data are much more restricted. In the course of this paper we refer to "cDNAs" as those transcript-derived sequences obtained from the above indicated repositories.

### cDNA-AFLP simulations

We simulated cDNA-AFLPs for all 28 combinations of eight different restriction enzymes for 126 pools of eukaryotic cDNA (105 species). The eight restriction enzymes used here are commonly used in AFLP screens and were used in a previous simulation study [6], allowing direct comparison with this earlier work. Enzyme details can be found in Table 4. Only restriction enzymes with 4-bp recognition sites were selected, as 6-bp restriction enzymes have been found to be ill-suited for cDNA-AFLP screens [[6], Kivioja, unpublished data]. We also collected information on the number of sequences and the sum of base pairs for each cDNA dataset and recorded nucleotide composition to estimate GC content and the proportion of non-ACGT base pairs (an indication of the overall quality of a dataset). The coverage of each cDNA pool was calculated as the percentage of cDNA transcripts which generated at least one fragment in the standard cDNA-AFLP size range (50 to 500 bp as commonly used on fluorescent sequencers) in an exhaustive PCR screen of all combinations of three selective base pairs. We termed this fraction "dataset coverage" and used it as our response variable.

Initial analyses revealed that a small number of cDNA datasets contained an unusually high proportion of non-ACGT nucleotides (>10%, data not shown). These datasets consisted of cDNA predictions based on early drafts of genome sequences for 13 mammalian species. Owing to the preliminary nature of these genome projects, many of the predicted cDNA sequences contained extended stretches of ambiguous base pairs ("Ns"). As a consequence, these sequences are effectively composed of two much shorter pieces of unambiguous sequence data. Because the probability of the presence of a particular restriction site is related to the length of a sequence, this reduction of the effective average sequence length can strongly influence the predicted cDNA pool coverage. As the peculiar nature of these poor quality datasets had a strong influence on preliminary GLMs, these species were excluded from further analyses. The remaining 113 datasets included here are listed in additional files 1 and 2.

Our simulations returned information for each dataset and enzyme combination in separate results files. This information was collated into summary files using EXCEL macros and a JAVA routine and imported into SAS 9.1.3. The *in silico *cDNA-AFLP routine, EXCEL macros, JAVA tool, and raw data sets are available upon request from the corresponding authors.

### Patterning of selective PCRs

Most AFLP studies use two or three selective base pairs in their selective PCRs. We produced the most inclusive arrays of selective *in silico *PCRs by counting fragment numbers produced for all possible combinations of selective PCRs with three selective base pairs for each dataset and restriction enzyme combination. Three selective base pairs for each selective primer allow for a maximum of 4^3 ^× 4^3 ^different primer combinations for two enzymes, and thus this most inclusive data array contains 4096 cells. Arrays for all species tested here are available in additional file 5. As some AFLP experiments use fewer selective base pairs, two-by-three and two-by-two selective base pair arrays were produced from the three-by-three array by summation. This summation is possible because the fragment numbers produced by amplifications with two selective base pairs are identical to those produced by all four selective amplifications obtained with three selective base pairs (ex: AAN for N = A, C, G, T), given that the first two selective base pairs are identical to those of the two base pair selective amplification. The two-by-three selective base pair arrays and the two-by-two selective base pair arrays contained 1024 and 256 cells, respectively.

We investigated the relative information content of all 4096 selective PCR reactions using graphical representations for a subset of PCR arrays. We also simulated DNA and cDNA datasets of 10000 sequences of 1290 bp using the SEQUENCE MANIPULATION SUITE[25]. Random DNA datasets were generated assuming equal base pair frequencies, while random cDNA datasets were generated using codon triplets based on the standard eukaryotic genetic code, starting with a start codon and ending with a stop codon. We compared these results with *in silico *cDNA-AFLP data derived from *Homo sapiens *(Figs. 2, 3). The same procedure was applied to the selective PCR arrays for six different species (Figure 4) to investigate systematic differences in cDNA-AFLP patterns across taxonomic groups. Data were visualized with the SAS/Graph bundle and the R library "Fields" [26].

### Partitioning variation in cDNA-pool coverage

Mixed model analyses (PROC MIXED) were used to study the relative importance of sequence characteristics and enzyme combinations in explaining cDNA pool coverage (an arcsine-square root transformed value unless otherwise indicated). All covariates were standardized by mean-centering and dividing by two standard deviations to control for the influence of different scaling factors in our predictor variables, and analyses were weighted by total pool size (in bp) to control for potential differences in variance estimates. We calculated partial R-square coefficients [27], which provide an indication of the strength of the influence of individual covariates on the response variable. Due to correlations between explanatory variables, these values do not necessarily sum to 100%. Complementary DNA pool coverage is expected to vary with the sequence characteristics of the underlying dataset, and average sequence length, GC content, the proportion of ambiguous nucleotides (non-ACGT) and total pool size were thus all included as covariates in our models.

Pools of complementary DNA were obtained from NCBI and ENSEMBL, two sequence repositories that use different methods for the organization and curation of their genetic data. As these differences could introduce an additional source of variation in our analyses, we investigated the importance of database origin, using the 21 taxa for which data were available from both repositories (see additional file 3). We modeled variation in cDNA pool coverage according to database origin, enzyme combination and the interactions of database origin and GC content with the enzyme combination (see additional file 4) in addition to the main effects of the covariates listed above. As coverage estimates for all 28 enzyme combinations were based on the same underlying cDNA pool for each species in each database, we controlled for species origin by incorporating a species (database) random effect. Because database origin did not explain a significant proportion of the variation in cDNA pool coverage after controlling for other covariates (see additional file 4), we removed duplicate species from the NCBI repository from further analyses to eliminate potential biases due to pseudoreplication.

### Effects of taxonomic grouping

Testing for the effect of taxonomic grouping (Table 5) was also possible, as 68 of the 92 available species could be assigned to eight taxonomic groups with three or more taxa using the NCBI Taxonomy browser [28]. Similar mixed models were used to investigate the effects of enzyme combination (Table 2; see additional file 1) and taxonomic grouping (Table 5; see additional file 2) on the cDNA pool coverage. These factors entered either analysis in addition to the covariates indicated above and the significant two-way interactions between GC content and enzyme combination/taxonomic group were retained in the final model. Here, we accounted for the nested nature of our data by including species as a random factor.

Because our mixed models estimate the combined effects of the two restriction enzymes, we isolated the individual effects of each restriction enzyme by regressing the untransformed cDNA pool coverage against individual restriction enzymes in a separate model (Table 4). Here, each of the eight restriction enzymes entered the model as dummy variables explaining variation in the cDNA pool coverage. In addition to the individual enzymes, we also included enzyme combination in the model to determine how much additional variation in coverage could be explained by enzyme interactions. As such, we were able to identify the separate effects of individual enzymes and their interactions on pool coverage. Parameter estimates from this linear regression are reported in Table 4, together with information on each restriction enzyme.

### Estimating underlying cDNA pool sizes by AFLP fragment number

Finally, we performed simple linear regression of the average fragment numbers per species and enzyme combination obtained during each selective PCR against the total size of the cDNA pool to explore whether the average number of fragments obtained per selective PCR provides information on the size of the underlying cDNA pool. This total pool size estimate can be directly transformed into an estimate of the total number of different cDNAs present in the studied pool by assuming an average sequence length of 1300-1400 bp [12]. By performing linear regressions of the average fragment numbers per selective PCR and enzyme combination for the 2 × 2, 2 × 3 and 3 × 3 arrays against the total cDNA pool size, we were able to determine the optimal number of selective base pairs for a given total pool size in order to minimize collisions (20 fragments per PCR, [8]), to optimize separation (50-100 fragments [3]) or to maximize the total number of fragments produced per selective PCR (up to 450 fragments can be scored over a typical AFLP screen of 50 to 500 bp). Figure 1 summarizes our findings and Table 3 reports regression coefficients and equations.

## Competing interests

The authors declare that they have no competing interests.

## Authors' contributions

KNS conceived of the study, collected raw data, executed statistical analyses and drafted the MS. GG programmed the analysis routine and was the primary impetus behind statistical analyses. CW programmed routines for the collation of raw data into single analysis files. ABW contributed to the conception of the study and the preparation of the MS and executed statistical analyses. All authors critically discussed results and conclusions and read and approved the final version of the manuscript.

## Supplementary Material

Additional file 1 **General information for each species**. General information for each of the 92 eukaryotic species included in the present study. Source identifies the database from which sequence pools were derived. The number of sequences included in each pool (N Seq) and the total pool size in base pairs (bp) are indicated. Avg Seq Lgt reports on the average sequence length, % GC indicates the percentage of GC nucleotides and Non-ACGT states the proportion of ambiguous nucleotides in each pool. Coverage ± SD reports the average percent coverage obtained across all 28 combinations of 8 tested restriction enzymes. The enzyme combination that provided the deepest cDNA pool coverage is indicated for each species. Click here for file

Additional file 2 **Species composition of included taxonomic groups**. Taxonomic groupings for the 68 eukaryotic species derived from eight taxonomic groups with three or more representatives. Tax group indicates the taxonomic group (according to NCBI Taxonomy browser). The number of sequences (N Seq), the total pool size (in base pairs), average sequence length (Avg Seq Lgt) and GC content (% GC) are shown. Average coverage (± SD), minimum and maximum coverage, along with the enzyme combination resulting in the deepest cDNA pool coverage for each species are indicated. Click here for file

Additional file 3 **Duplicate species from ENSEMBL and NCBI databases**. Duplicate species from the ENSEMBL and NCBI databases. Average sequence length (Avg Seq Lgt), organismal GC-content (% GC) and the percentage of ambiguous base pairs (% non-ACGT) are indicated. The average pool coverage per enzyme combination, along with maximum and minimum coverage values, are shown. Click here for file

Additional file 4 **Influence of database origin on pool coverage**. The influence of database origin and enzyme choice on cDNA pool coverage for the 21 species present in both databases. We accounted for variability in coverage resulting from the nesting of species within database and weighted cDNA pool coverage by the number of sequences per pool to account for variation in available sequence data. Denominator degrees of freedom were Kenward-Roger corrected. Partial R-square indicates the proportion of the variation in cDNA pool coverage which is explained by each factor/interaction [24]. Click here for file

Additional file 5 **Arrays of all selective PCRs for all species and enzyme combinations**. Selective PCR arrays using three-by-three selective base pairs for all 87 NCBI and 39 ENSEMBL species included in the present study, tested on all 28 combinations of 8 restriction enzymes. Click here for file
