# Supplementary material for: Eukaryotic transcriptomics in silico: Optimizing cDNA-AFLP efficiency
Source: BMC Genomics. 2009 Nov 30;10:565. doi: 10.1186/1471-2164-10-565 (PMC2797533; doi:10.1186/1471-2164-10-565)
Supplement: Additional file 1 — General information for each species. General information for each of the 92 eukaryotic species included in the present study. Source identifies the database from which sequence pools were derived. The number of sequences included in each pool (N Seq) and the total pool size in base pairs (bp) are indicated. Avg Seq Lgt reports on the average sequence length, % GC indicates the percentage of GC nucleotides and Non-ACGT states the proportion of ambiguous nucleotides in each pool. Coverage ± SD reports the average percent coverage obtained across all 28 combinations of 8 tested restriction enzymes. The enzyme combination that provided the deepest cDNA pool coverage is indicated for each species. [file 1471-2164-10-565-S1.DOC]

Additional file 1 - General information for each species

General information for each of the 92 eukaryotic species included in the present study. Source identifies the database from which sequence pools were derived. The number of sequences included in each pool (N Seq) and the total pool size in base pairs (bp) are indicated. Avg Seq Lgt reports on the average sequence length, % GC indicates the percentage of GC nucleotides and Non-ACGT states the proportion of ambiguous nucleotides in each pool. Coverage ± SD reports the average percent coverage obtained across all 28 combinations of 8 tested restriction enzymes. The enzyme combination that provided the deepest cDNA pool coverage is indicated for each species.

| **Species** | **Source** | **N Seq** | **Total pool size (bp)** | **Avg Seq Lgt** | **% GC** | **Non-ACGT** | **Coverage ± SD** | **Min-Max coverage** | **Best Combination** |
| --- | --- | --- | --- | --- | --- | --- | --- | --- | --- |
| *Acrythosiphon pisum* | NCBI | 6557 | 4044893 | 616.88 | 33.09 | 0.02 | 38.73 ± 15.71 | 16.03 - 70.95 | MseI - CviQI |
| *Aedes aegypti* | ENSEMBL | 18061 | 27616123 | 1529.05 | 47.98 | 0.02 | 74.92 ± 7.17 | 59.49 - 85.72 | HpaII - TaqI |
| *Anopheles gambiae* | ENSEMBL | 13133 | 20879537 | 1589.85 | 55.26 | 0.00 | 78.05 ± 12.71 | 49.41 - 92.32 | TaqI - CviQI |
| *Apis mellifera* | NCBI | 9791 | 13386956 | 1367.27 | 37.15 | 0.02 | 54.04 ± 15.89 | 28.75 - 79.91 | MseI - TaqI |
| *Aquilegia formosa x pubescens* | NCBI | 8065 | 7359417 | 912.51 | 41.00 | 0.00 | 51.88 ± 21.58 | 17.15 - 91.97 | MseI - CviAII |
| *Arabidopsis thaliana* | NCBI | 29974 | 43315662 | 1445.11 | 42.16 | 0.02 | 67.76 ± 14.26 | 41.39 - 88.45 | MseI - CviAII |
| *Bombyx mori* | NCBI | 9939 | 7662004 | 770.90 | 39.02 | 0.10 | 56.14 ± 11.90 | 35.87 - 77.49 | MseI - CviQI |
| *Bos taurus* | ENSEMBL | 28958 | 49808680 | 1720.03 | 52.50 | 0.01 | 61.64 ± 7.63 | 49.56 - 79.20 | CviAII - CviQI |
| *Branchiostoma floridae* | NCBI | 11507 | 8085133 | 702.63 | 41.81 | 0.25 | 45.71 ± 17.69 | 23.53 - 84.70 | CviAII - CviQI |
| *Brassica napus* | NCBI | 26287 | 20322912 | 773.12 | 44.99 | 0.17 | 59.90 ± 11.75 | 38.83 - 80.12 | MseI - CviAII |
| *Caenorhabditis elegans* | ENSEMBL | 28981 | 40353676 | 1392.42 | 42.34 | 0.00 | 63.29 ± 13.18 | 34.74 - 84.00 | CviAII - TaqI |
| *Canis familiaris* | ENSEMBL | 27301 | 42169482 | 1544.61 | 51.89 | 0.00 | 59.50 ± 8.84 | 46.10 - 80.16 | CviAII - CviQI |
| *Chlamydomonas reinhardtii* | NCBI | 11276 | 16939170 | 1502.23 | 63.71 | 0.02 | 73.22 ± 16.24 | 40.40 - 95.05 | HinP1I - CviAII |
| *Ciona intestinalis* | ENSEMBL | 19858 | 29064597 | 1463.62 | 41.60 | 0.00 | 64.88 ± 13.73 | 39.04 - 90.20 | MseI - CviAII |
| *Ciona savignyi* | ENSEMBL | 20359 | 32691732 | 1605.76 | 44.98 | 0.00 | 71.86 ± 10.99 | 48.09 - 88.73 | MseI - CviAII |
| *Citrus clementina* | NCBI | 6107 | 6788422 | 1111.58 | 44.43 | 0.06 | 63.58 ± 11.26 | 44.20 - 88.83 | MseI - CviAII |
| *Citrus sinensis* | NCBI | 9699 | 7370218 | 759.89 | 41.84 | 0.06 | 48.11 ± 14.69 | 25.29 - 82.75 | MseI - CviAII |
| *Coccidioides posadasii* | NCBI | 3994 | 3776554 | 945.56 | 49.82 | 0.00 | 76.32 ± 8.47 | 60.49 - 93.54 | CviAII - TaqI |
| *Danio rerio* | ENSEMBL | 31841 | 51017126 | 1602.25 | 48.09 | 0.00 | 67.11 ± 8.90 | 50.64 - 84.63 | MseI - CviAII |
| *Dictyostelium discoideum* | NCBI | 5960 | 4195845 | 704.00 | 29.36 | 0.29 | 22.42 ± 19.10 | 1.36 - 65.32 | MseI - CviAII |
| *Drosophila melanogaster* | ENSEMBL | 20909 | 48315668 | 2310.76 | 49.91 | 0.00 | 84.67 ± 6.18 | 71.79 - 92.33 | CviAII - TaqI |
| *Equus caballus* | ENSEMBL | 27192 | 46568281 | 1712.57 | 50.03 | 0.00 | 56.74 ± 8.04 | 42.77 - 75.27 | CviAII - CviQI |
| *Felis catus* | ENSEMBL | 15993 | 20792059 | 1300.07 | 53.89 | 0.00 | 54.92 ± 9.45 | 39.87 - 74.44 | HpaII - CviAII |
| *Filobasidiella neoformans* | NCBI | 3559 | 7052090 | 1981.48 | 50.33 | 0.00 | 86.26 ± 6.26 | 70.86 - 96.94 | CviAII - TaqI |
| *Fundulus heteroclitus* | NCBI | 4573 | 3367792 | 736.45 | 47.39 | 0.55 | 48.34 ± 12.03 | 25.39 - 72.12 | MseI - CviAII |
| *Gadus morhua* | NCBI | 10792 | 7919862 | 733.86 | 43.20 | 0.04 | 52.86 ± 13.68 | 30.47 - 84.51 | MseI - CviAII |
| *Gallus gallus* | ENSEMBL | 22291 | 39792561 | 1785.14 | 48.61 | 0.00 | 61.38 ± 9.22 | 43.79 - 81.48 | CviAII - CviQI |
| *Gasterosteus aculeatus* | ENSEMBL | 27629 | 45847847 | 1659.41 | 55.02 | 0.00 | 70.69 ± 15.89 | 39.48 - 89.39 | HpaII - CviAII |
| *Gibberella moniliformis* | NCBI | 5259 | 4752592 | 903.71 | 51.71 | 0.00 | 68.29 ± 13.27 | 41.51 - 93.61 | CviAII - TaqI |
| *Glycine max* | NCBI | 24518 | 17344657 | 707.43 | 41.21 | 0.43 | 40.93 ± 16.26 | 17.90 - 80.96 | MseI - CviAII |
| *Gossypium hirsutum* | NCBI | 16404 | 12887278 | 785.62 | 42.98 | 0.05 | 51.70 ± 16.22 | 24.66 - 85.10 | MseI - CviAII |
| *Gossypium raimondii* | NCBI | 3295 | 2698120 | 818.85 | 43.93 | 0.02 | 53.46 ± 17.15 | 23.70 - 87.95 | MseI - CviAII |
| *Helianthus annuus* | NCBI | 7969 | 5407728 | 678.60 | 42.80 | 0.07 | 50.47 ± 13.71 | 27.42 - 79.57 | MseI - CviAII |
| *Homo sapiens* | ENSEMBL | 48803 | 125500000 | 2571.64 | 49.68 | 0.00 | 71.73 ± 6.9 | 61.95 - 85.59 | CviAII - CviQI |
| *Hordeum vulgare* | NCBI | 22853 | 20147314 | 881.60 | 51.19 | 0.22 | 63.14 ± 7.96 | 49.55 - 79.50 | CviAII - TaqI |
| *Hydra magnipapillata* | NCBI | 10923 | 7092578 | 649.33 | 32.88 | 0.05 | 35.29 ± 17.12 | 9.79 - 75.38 | MseI - CviAII |
| *Lactuca sativa* | NCBI | 7848 | 6566967 | 836.77 | 42.92 | 0.05 | 54.32 ± 16.68 | 27.13 - 86.96 | MseI - CviAII |
| *Lotus japonicus* | NCBI | 13659 | 7282469 | 533.16 | 42.23 | 0.11 | 33.12 ± 13.89 | 14.84 - 71.72 | MseI - CviAII |
| *Macaca fascicularis* | NCBI | 10799 | 17252467 | 1597.60 | 45.93 | 0.04 | 64.36 ± 11.38 | 46.16 - 88.40 | MseI - CviAII |
| *Macaca mulatta* | ENSEMBL | 38146 | 70430633 | 1846.34 | 50.36 | 0.01 | 62.24 ± 7.45 | 52.42 - 78.74 | CviAII - CviQI |
| *Malus x domestica* | NCBI | 16913 | 10632914 | 628.68 | 44.88 | 0.07 | 46.35 ± 11.43 | 27.74 - 70.45 | MseI - CviAII |
| *Medicago truncatula* | NCBI | 17785 | 12924130 | 726.69 | 39.72 | 0.39 | 43.07 ± 17.13 | 17.03 - 83.13 | MseI - CviAII |
| *Meleagris gallopavo* | NCBI | 960 | 679555 | 707.87 | 47.42 | 0.08 | 38.07 ± 12.05 | 17.71 - 69.48 | CviAII - CviQI |
| *Molgula tectiformis* | NCBI | 8534 | 6725171 | 788.04 | 35.42 | 0.32 | 47.35 ± 17.86 | 21.69 - 88.73 | MseI - CviAII |
| *Monodelphis domestica* | ENSEMBL | 33279 | 57497869 | 1727.75 | 48.10 | 0.00 | 58.51 ± 11.68 | 38.49 - 83.06 | CviAII - CviQI |
| *Mus musculus* | ENSEMBL | 40959 | 99678366 | 2433.61 | 49.98 | 0.00 | 71.96 ± 7.36 | 60.58 - 87.00 | CviAII - CviQI |
| *Neurospora crassa* | NCBI | 2209 | 1269530 | 574.71 | 51.57 | 0.13 | 48.56 ± 11.72 | 27.61 - 71.48 | CviAII - TaqI |
| *Nicotiana tabacum* | NCBI | 13207 | 10073670 | 762.75 | 41.46 | 0.28 | 46.96 ± 15.63 | 21.71 - 80.91 | MseI - CviAII |
| *Oncorhynchus mykiss* | NCBI | 25264 | 21635734 | 856.39 | 45.10 | 0.60 | 50.12 ± 14.27 | 28.64 - 82.10 | MseI - CviAII |
| *Ornithorhynchus anatinus* | ENSEMBL | 27383 | 37194655 | 1358.31 | 53.33 | 0.01 | 59.13 ± 10.3 | 40.07 - 79.33 | HpaII - CviAII |
| *Oryctolagus cuniculus* | NCBI | 6517 | 5377786 | 825.19 | 49.96 | 0.02 | 39.71 ± 8.82 | 26.21 - 61.49 | MseI - CviAII |
| *Oryza sativa* | NCBI | 40742 | 62731750 | 1539.73 | 50.75 | 0.09 | 72.75 ± 6.63 | 60.58 - 86.03 | CviAII - TaqI |
| *Oryzias latipes* | ENSEMBL | 24662 | 38325234 | 1554.02 | 52.36 | 0.01 | 65.66 ± 12.19 | 44.21 - 82.86 | HpaII - CviAII |
| *Ovis aries* | NCBI | 12195 | 9682040 | 793.94 | 50.36 | 0.01 | 46.76 ± 9.72 | 32.96 - 67.61 | CviAII - CviQI |
| *Pan troglodytes* | ENSEMBL | 34009 | 78022597 | 2294.17 | 49.50 | 0.01 | 68.47 ± 7.09 | 59.02 - 83.68 | CviAII - CviQI |
| *Paracentrotus lividus* | NCBI | 8664 | 7473899 | 862.64 | 43.09 | 0.31 | 54.75 ± 15.93 | 25.67 - 83.55 | CviAII - CviQI |
| *Paramecium tetraurelia* | NCBI | 14325 | 18863127 | 1316.80 | 31.58 | 0.13 | 34.66 ± 27.14 | 1.85 - 84.64 | MseI - CviAII |
| *Petromyzon marinus* | NCBI | 8512 | 5620756 | 660.33 | 47.53 | 0.01 | 55.32 ± 11.29 | 33.13 - 74.02 | MseI - CviAII |
| *Phaeodactylum tricornutum* | NCBI | 6772 | 6808242 | 1005.35 | 50.42 | 0.08 | 77.59 ± 12.06 | 49.79 - 92.76 | HpaII - TaqI |
| *Physcomitrella patens* | NCBI | 17973 | 14198438 | 789.99 | 47.78 | 0.10 | 63.70 ± 8.68 | 50.87 - 85.28 | CviAII - TaqI |
| *Phytophtora infestans* | NCBI | 7270 | 4996923 | 687.33 | 52.75 | 0.39 | 70.99 ± 9.98 | 51.38 - 85.24 | CviAII - TaqI |
| *Picea glauca* | NCBI | 17812 | 13565571 | 761.60 | 40.63 | 0.64 | 45.90 ± 16.11 | 23.70 - 85.03 | MseI – CviAII |
| *Picea sitchensis* | NCBI | 15699 | 11905367 | 758.35 | 42.55 | 0.00 | 48.28 ± 13.53 | 29.91 - 80.79 | MseI - CviAII |
| *Pimephales promelas* | NCBI | 22442 | 17900531 | 797.64 | 44.30 | 0.04 | 51.01 ± 12.26 | 31.59 - 83.62 | MseI - CviAII |
| *Pinus taeda* | NCBI | 18938 | 15068523 | 795.68 | 43.97 | 0.14 | 52.19 ± 12.79 | 33.39 - 82.41 | MseI - CviAII |
| *Pongo pygmaeus* | ENSEMBL | 24431 | 43871572 | 1795.73 | 50.25 | 0.01 | 58.88 ± 7.50 | 47.64 - 75.85 | CviAII - CviQI |
| *Populus balsamifera* | NCBI | 11310 | 8229010 | 727.59 | 41.07 | 0.00 | 44.05 ± 16.84 | 19.09 - 83.79 | MseI - CviAII |
| *Populus tremula x tremuloides* | NCBI | 7853 | 4925686 | 627.24 | 42.47 | 0.04 | 41.13 ± 15.09 | 17.80 - 76.91 | MseI - CviAII |
| *Populus trichocarpa* | NCBI | 14059 | 10473626 | 744.98 | 40.93 | 0.00 | 44.54 ± 17.14 | 18.67 - 84.12 | MseI - CviAII |
| *Prunus persica* | NCBI | 7062 | 4677442 | 662.34 | 42.50 | 0.12 | 41.86 ± 13.31 | 21.81 - 74.45 | MseI - CviAII |
| *Rattus norvegicus* | ENSEMBL | 34704 | 60508280 | 1743.55 | 51.10 | 0.01 | 62.60 ± 7.74 | 51.83 - 81.13 | CviAII - CviQI |
| *Saccharomyces cerevisiae* | ENSEMBL | 6698 | 9056373 | 1352.10 | 39.61 | 0.00 | 64.49 ± 11.14 | 38.85 - 80.01 | MseI - CviAII |
| *Saccharum officinarum* | NCBI | 15592 | 12706205 | 814.92 | 50.56 | 0.36 | 61.23 ± 8.57 | 44.90 - 77.28 | CviAII - TaqI |
| *Salmo salar* | NCBI | 29722 | 22077976 | 742.82 | 43.34 | 0.01 | 44.02 ± 16.83 | 21.51 - 82.16 | MseI - CviAII |
| *Schistosoma japonicum* | NCBI | 9107 | 8309606 | 912.44 | 35.01 | 0.09 | 55.09 ± 21.10 | 18.79 - 87.44 | MseI - CviAII |
| *Schistosoma mansoni* | NCBI | 9172 | 5822751 | 634.84 | 36.94 | 0.20 | 45.46 ± 19.22 | 14.11 - 75.24 | MseI - CviAII |
| *Solanum lycopersicum* | NCBI | 17849 | 15412230 | 863.48 | 40.55 | 0.27 | 47.90 ± 16.61 | 20.62 - 81.69 | MseI - CviAII |
| *Solanum tuberosum* | NCBI | 19671 | 15691567 | 797.70 | 40.98 | 0.02 | 48.51 ± 16.99 | 21.89 - 83.79 | MseI - CviAII |
| *Sorghum bicolor* | NCBI | 13984 | 9709132 | 694.30 | 51.83 | 0.02 | 55.05 ± 9.62 | 37.25 - 74.96 | CviAII - TaqI |
| *Strongylocentrus purpuratus* | NCBI | 19625 | 28042628 | 1428.92 | 43.49 | 0.25 | 62.47 ± 13.54 | 39.96 - 88.61 | MseI - CviAII |
| *Sus scrofa* | NCBI | 51706 | 42874695 | 829.20 | 47.29 | 0.09 | 45.12 ± 10.95 | 31.94 - 73.95 | MseI - CviAII |
| *Taeniopygia guttata* | NCBI | 11227 | 8347852 | 743.55 | 44.55 | 0.31 | 34.33 ± 15.64 | 17.67 - 77.69 | MseI - CviAII |
| *Takifugu rubripes* | ENSEMBL | 48027 | 91791931 | 1911.26 | 53.96 | 0.00 | 72.10 ± 14.78 | 43.41 - 88.88 | HpaII - CviAII |
| *Tetraodon nigroviridis* | ENSEMBL | 27991 | 37821073 | 1351.19 | 55.10 | 0.43 | 55.53 ± 13.30 | 30.89 - 73.81 | HpaII - CviAII |
| *Toxoplasma gondii* | NCBI | 6623 | 4448416 | 671.66 | 52.16 | 0.16 | 52.63 ± 13.45 | 27.92 - 74.47 | HinP1I - TaqI |
| *Tribolium castaneum* | NCBI | 9013 | 12745306 | 1414.10 | 44.63 | 0.62 | 69.83 ± 7.73 | 52.41 - 82.41 | HpaII - MseI |
| *Trichosurus vulpecula* | NCBI | 11757 | 9654352 | 821.16 | 40.68 | 0.73 | 36.91 ± 22.28 | 11.89 - 86.68 | MseI - CviAII |
| *Triticum aestivum* | NCBI | 41358 | 31737158 | 767.38 | 50.47 | 0.86 | 60.12 ± 7.92 | 46.51 - 76.64 | CviAII - TaqI |
| *Vitis vinifera* | NCBI | 23129 | 17999693 | 778.23 | 42.64 | 0.14 | 45.08 ± 14.72 | 22.05 - 80.78 | MseI - CviAII |
| *Xenopus laevis* | NCBI | 35518 | 45020703 | 1267.55 | 42.92 | 0.54 | 54.19 ± 13.31 | 34.31 - 86.97 | MseI - CviAII |
| *Xenopus tropicalis* | ENSEMBL | 27711 | 45111427 | 1627.92 | 46.38 | 0.00 | 62.21 ± 9.92 | 47.74 - 84.39 | CviAII - CviQI |
| *Zea mays* | NCBI | 57495 | 32228704 | 560.55 | 50.58 | 0.52 | 38.74 ± 4.90 | 29.81 - 48.70 | CviAII - TaqI |
