# Supplementary material for: Eukaryotic transcriptomics in silico: Optimizing cDNA-AFLP efficiency
Source: BMC Genomics. 2009 Nov 30;10:565. doi: 10.1186/1471-2164-10-565 (PMC2797533; doi:10.1186/1471-2164-10-565)
Supplement: Additional file 2 — Species composition of included taxonomic groups. Taxonomic groupings for the 68 eukaryotic species derived from eight taxonomic groups with three or more representatives. Tax group indicates the taxonomic group (according to NCBI Taxonomy browser). The number of sequences (N Seq), the total pool size (in base pairs), average sequence length (Avg Seq Lgt) and GC content (% GC) are shown. Average coverage (± SD), minimum and maximum coverage, along with the enzyme combination resulting in the deepest cDNA pool coverage for each species are indicated. [file 1471-2164-10-565-S2.DOC]

Additional file 2 - Species composition of taxonomic groups

Taxonomic groupings for the 68 eukaryotic species derived from eight taxonomic groups with three or more representatives. Tax group indicates the taxonomic group (according to Ncbi Taxonomy browser). The number of sequences (N Seq), the total pool size (in base pairs), average sequence length (Avg Seq Lgt) and GC content (% GC) are shown. Average coverage (± SD), minimum and maximum coverage, along with the enzyme combination resulting in the deepest cDNA pool coverage for each species are indicated.

| **Tax Group** | **Species** | **Source** | **N Seq** | **Total pool size (bp)** | **Avg Seq Lgt** | **% GC** | **Coverage ± SD** | **Min-Max Cov.** | **Best Combination** |
| --- | --- | --- | --- | --- | --- | --- | --- | --- | --- |
| Actinopterygii | *Danio rerio* | ENSEMBL | 31841 | 51017126 | 1602.25 | 48.09 | 67.11 ± 8.90 | 50.64 - 84.63 | MseI - CviAII |
| Actinopterygii | *Fundulus heteroclitus* | NCBI | 4573 | 3367792 | 736.45 | 47.39 | 48.34 ± 12.03 | 25.39 - 72.12 | MseI - CviAII |
| Actinopterygii | *Gadus morhua* | NCBI | 10792 | 7919862 | 733.86 | 43.20 | 52.86 ± 13.69 | 30.47 - 84.51 | MseI - CviAII |
| Actinopterygii | *Gasterosteus aculeatus* | ENSEMBL | 27629 | 45847847 | 1659.41 | 55.02 | 70.69 ± 15.89 | 39.48 - 89.39 | HpaII - CviAII |
| Actinopterygii | *Oncorhynchus mykiss* | NCBI | 25264 | 21635734 | 856.39 | 45.10 | 50.12 ± 14.27 | 28.64 - 82.10 | MseI - CviAII |
| Actinopterygii | *Oryzias latipes* | ENSEMBL | 24662 | 38325234 | 1554.02 | 52.36 | 65.66 ± 12.19 | 44.21 - 82.86 | HpaII - CviAII |
| Actinopterygii | *Pimephales promelas* | NCBI | 22442 | 17900531 | 797.64 | 44.30 | 51.01 ± 12.26 | 31.59 - 83.62 | MseI - CviAII |
| Actinopterygii | *Salmo salar* | NCBI | 29722 | 22077976 | 742.82 | 43.34 | 44.02 ± 16.83 | 21.51 - 82.16 | MseI - CviAII |
| Actinopterygii | *Takifugu rubripes* | ENSEMBL | 48027 | 91791931 | 1911.26 | 53.96 | 72.10 ± 14.78 | 43.41 - 88.88 | HpaII - CviAII |
| Actinopterygii | *Tetraodon nigroviridis* | ENSEMBL | 27991 | 37821073 | 1351.19 | 55.10 | 55.53 ± 13.30 | 30.89 - 73.81 | HpaII - CviAII |
| **Avg. Actinopterygii (N=10)** | | | **25294** | **33770511** | **1194.53** | **48.79** | **57.74 ± 16.50** | **21.51 - 89.39** |  |
| Ascidiaceae | *Ciona intestinalis* | ENSEMBL | 19858 | 29064597 | 1463.62 | 41.60 | 64.88 ± 13.73 | 39.04 - 90.20 | MseI - CviAII |
| Ascidiaceae | *Ciona savignyi* | ENSEMBL | 20359 | 32691732 | 1605.76 | 44.98 | 71.86 ± 10.99 | 48.09 - 88.73 | MseI - CviAII |
| Ascidiaceae | *Molgula tectiformis* | NCBI | 8534 | 6725171 | 788.04 | 35.42 | 47.35 ± 17.86 | 21.69 - 88.73 | MseI - CviAII |
| **Avg. Ascidiaceae (N=3)** | | | **16250** | **22827167** | **1285.81** | **40.67** | **61.36 ± 17.66** | **21.69 - 90.20** |  |
| Aves | *Gallus gallus* | ENSEMBL | 22291 | 39792561 | 1785.14 | 48.61 | 61.38 ± 9.22 | 43.79 - 81.48 | CviAII - CviQI |
| Aves | *Meleagris gallopavo* | NCBI | 960 | 679555 | 707.87 | 47.42 | 38.07 ± 12.05 | 17.71 - 69.48 | CviAII - CviQI |
| Aves | *Taeniopygia guttata* | NCBI | 11227 | 8347852 | 743.55 | 44.55 | 34.33 ± 15.64 | 17.67 - 77.69 | MseI - CviAII |
| **Avg. Aves (N=3)** | | | **11493** | **16273323** | **1078.85** | **46.86** | **44.59 ± 17.31** | **17.67 - 81.48** |  |
| Coniferopsida | *Picea glauca* | NCBI | 17812 | 13565571 | 761.6 | 40.63 | 45.90 ± 16.11 | 23.70 - 85.03 | MseI - CviAII |
| Coniferopsida | *Picea sitchensis* | NCBI | 15699 | 11905367 | 758.35 | 42.55 | 48.28 ± 13.53 | 29.91 - 80.79 | MseI - CviAII |
| Coniferopsida | *Pinus taeda* | NCBI | 18938 | 15068523 | 795.68 | 43.97 | 52.19 ± 12.79 | 33.39 - 82.41 | MseI - CviAII |
| **Avg. Coniferopsida (N=3)** | | | **17483** | **13513154** | **771.88** | **42.38** | **48.79 ± 14.29** | **23.70 - 85.03** |  |
| Insecta | *Acrythosiphon pisum* | NCBI | 6557 | 4044893 | 616.88 | 33.09 | 38.73 ± 15.71 | 16.03 - 70.95 | MseI - CviQI |
| Insecta | *Aedes aegypti* | ENSEMBL | 18061 | 27616123 | 1529.05 | 47.98 | 74.92 ± 7.17 | 59.49 - 85.72 | HpaII - TaqI |
| Insecta | *Anopheles gambiae* | ENSEMBL | 13133 | 20879537 | 1589.85 | 55.26 | 78.05 ± 12.71 | 49.41 - 92.32 | TaqI - CviQI |
| Insecta | *Apis mellifera* | NCBI | 9791 | 13386956 | 1367.27 | 37.15 | 54.04 ± 15.89 | 28.75 - 79.91 | MseI - TaqI |
| Insecta | *Bombyx mori* | NCBI | 9939 | 7662004 | 770.9 | 39.02 | 56.14 ± 11.90 | 35.87 - 77.49 | MseI - CviQI |
| Insecta | *Drosophila melanogaster* | ENSEMBL | 20909 | 48315668 | 2310.76 | 49.91 | 84.67 ± 6.18 | 71.79 - 92.33 | CviAII - TaqI |
| Insecta | *Tribolium castaneum* | NCBI | 9013 | 12745306 | 1414.1 | 44.63 | 69.83 ± 7.73 | 52.41 - 82.41 | HpaII - MseI |
| **Avg. Insecta (N=7)** | | | **12486** | **19235784** | **1371.26** | **43.86** | **65.20 ± 18.89** | **16.03 - 92.33** |  |
| Liliopsida | *Hordeum vulgare* | NCBI | 22853 | 20147314 | 881.6 | 51.19 | 63.14 ± 7.96 | 49.55 - 79.50 | CviAII - TaqI |
| Liliopsida | *Oryza sativa* | NCBI | 40742 | 62731750 | 1539.73 | 50.75 | 72.75 ± 6.63 | 60.58 - 86.03 | CviAII - TaqI |
| Liliopsida | *Saccharum officinarum* | NCBI | 15592 | 12706205 | 814.92 | 50.56 | 61.23 ± 8.57 | 44.90 - 77.28 | CviAII - TaqI |
| Liliopsida | *Sorghum bicolor* | NCBI | 13984 | 9709132 | 694.3 | 51.83 | 55.05 ± 9.62 | 37.25 - 74.96 | CviAII - TaqI |
| Liliopsida | *Triticum aestivum* | NCBI | 41358 | 31737158 | 767.38 | 50.47 | 60.12 ± 7.92 | 46.51 - 76.64 | CviAII - TaqI |
| Liliopsida | *Zea mays* | NCBI | 57495 | 32228704 | 560.55 | 50.58 | 38.74 ± 4.90 | 29.81 - 48.70 | CviAII - TaqI |
| **Avg. Liliopsida (N=6)** | | | **32004** | **28210044** | **876.41** | **50.90** | **58.51 ± 12.85** | **29.81 - 86.03** |  |
| Mammalia | *Bos taurus* | ENSEMBL | 28958 | 49808680 | 1720.03 | 52.50 | 61.64 ± 7.63 | 49.56 - 79.20 | CviAII - CviQI |
| Mammalia | *Canis familiaris* | ENSEMBL | 27301 | 42169482 | 1544.61 | 51.89 | 59.50 ± 8.84 | 46.10 - 80.16 | CviAII - CviQI |
| Mammalia | *Equus caballus* | ENSEMBL | 27192 | 46568281 | 1712.57 | 50.03 | 56.74 ± 8.04 | 42.77 - 75.27 | CviAII - CviQI |
| Mammalia | *Felis catus* | ENSEMBL | 15993 | 20792059 | 1300.07 | 53.89 | 54.92 ± 9.45 | 39.87 - 74.44 | HpaII - CviAII |
| Mammalia | *Homo sapiens* | ENSEMBL | 48803 | 125500000 | 2571.64 | 49.68 | 71.73 ± 6.90 | 61.95 - 85.59 | CviAII - CviQI |
| Mammalia | *Macaca fascicularis* | NCBI | 10799 | 17252467 | 1597.6 | 45.93 | 64.36 ± 11.38 | 46.16 - 88.40 | MseI - CviAII |
| Mammalia | *Macaca mulatta* | ENSEMBL | 38146 | 70430633 | 1846.34 | 50.36 | 62.24 ± 7.45 | 52.42 - 78.74 | CviAII - CviQI |
| Mammalia | *Monodelphis domestica* | ENSEMBL | 33279 | 57497869 | 1727.75 | 48.10 | 58.51 ± 11.68 | 38.49 - 83.06 | CviAII - CviQI |
| Mammalia | *Mus musculus* | ENSEMBL | 40959 | 99678366 | 2433.61 | 49.98 | 71.96 ± 7.36 | 60.58 – 87.00 | CviAII - CviQI |
| Mammalia | *Ornithorhynchus anatinus* | ENSEMBL | 27383 | 37194655 | 1358.31 | 53.33 | 59.13 ± 10.30 | 40.07 - 79.33 | HpaII - CviAII |
| Mammalia | *Oryctolagus cuniculus* | NCBI | 6517 | 5377786 | 825.19 | 49.96 | 39.71 ± 8.82 | 26.21 - 61.49 | MseI - CviAII |
| Mammalia | *Ovis aries* | NCBI | 12195 | 9682040 | 793.94 | 50.36 | 46.76 ± 9.72 | 32.96 - 67.61 | CviAII - CviQI |
| Mammalia | *Pan troglodytes* | ENSEMBL | 34009 | 78022597 | 2294.17 | 49.50 | 68.47 ± 7.09 | 59.02 - 83.68 | CviAII - CviQI |
| Mammalia | *Pongo pygmaeus* | ENSEMBL | 24431 | 43871572 | 1795.73 | 50.25 | 58.88 ± 7.50 | 47.64 - 75.85 | CviAII - CviQI |
| Mammalia | *Rattus norvegicus* | ENSEMBL | 34704 | 60508280 | 1743.55 | 51.10 | 62.60 ± 7.74 | 51.83 - 81.13 | CviAII - CviQI |
| Mammalia | *Sus scrofa* | NCBI | 51706 | 42874695 | 829.2 | 47.29 | 45.12 ± 10.95 | 31.94 - 73.95 | MseI - CviAII |
| Mammalia | *Trichosurus vulpecula* | NCBI | 11757 | 9654352 | 821.16 | 40.68 | 36.91 ± 22.28 | 11.89 - 86.68 | MseI - CviAII |
| **Avg. Mammalia (N=17)** | | | **27890** | **48051989** | **1583.26** | **49.70** | **57.60 ± 14.12** | **11.89 - 88.40** |  |
| Streptophyta | *Arabidopsis thaliana* | NCBI | 29974 | 43315662 | 1445.11 | 42.16 | 67.76 ± 14.26 | 41.39 - 88.45 | MseI - CviAII |
| Streptophyta | *Brassica napus* | NCBI | 26287 | 20322912 | 773.12 | 44.99 | 59.90 ± 11.75 | 38.83 - 80.12 | MseI - CviAII |
| Streptophyta | *Citrus clementina* | NCBI | 6107 | 6788422 | 1111.58 | 44.43 | 63.58 ± 11.26 | 44.20 - 88.83 | MseI - CviAII |
| Streptophyta | *Citrus sinensis* | NCBI | 9699 | 7370218 | 759.89 | 41.84 | 48.11 ± 14.69 | 25.29 - 82.75 | MseI - CviAII |
| Streptophyta | *Glycine max* | NCBI | 24518 | 17344657 | 707.43 | 41.21 | 40.94 ± 16.26 | 17.90 - 80.96 | MseI - CviAII |
| Streptophyta | *Gossypium hirsutum* | NCBI | 16404 | 12887278 | 785.62 | 42.98 | 51.70 ± 16.22 | 24.66 - 85.10 | MseI - CviAII |
| Streptophyta | *Gossypium raimondii* | NCBI | 3295 | 2698120 | 818.85 | 43.93 | 53.46 ± 17.15 | 23.70 - 87.95 | MseI - CviAII |
| Streptophyta | *Helianthus annuus* | NCBI | 7969 | 5407728 | 678.6 | 42.80 | 50.47 ± 13.71 | 27.42 - 79.57 | MseI - CviAII |
| Streptophyta | *Lactuca sativa* | NCBI | 7848 | 6566967 | 836.77 | 42.92 | 54.32 ± 16.68 | 27.13 - 86.96 | MseI - CviAII |
| Streptophyta | *Lotus japonicus* | NCBI | 13659 | 7282469 | 533.16 | 42.23 | 33.12 ± 13.89 | 14.84 - 71.72 | MseI - CviAII |
| Streptophyta | *Malus x domestica* | NCBI | 16913 | 10632914 | 628.68 | 44.88 | 46.35 ± 11.43 | 27.74 - 70.45 | MseI - CviAII |
| Streptophyta | *Medicago truncatula* | NCBI | 17785 | 12924130 | 726.69 | 39.72 | 43.07 ± 17.13 | 17.03 - 83.13 | MseI - CviAII |
| Streptophyta | *Nicotiana tabacum* | NCBI | 13207 | 10073670 | 762.75 | 41.46 | 46.96 ± 15.63 | 21.71 - 80.91 | MseI - CviAII |
| Streptophyta | *Populus balsamifera* | NCBI | 11310 | 8229010 | 727.59 | 41.07 | 44.05 ± 16.84 | 19.09 - 83.79 | MseI - CviAII |
| Streptophyta | *Populus tremula x tremuloides* | NCBI | 7853 | 4925686 | 627.24 | 42.47 | 41.13 ± 15.10 | 17.80 - 76.91 | MseI - CviAII |
| Streptophyta | *Populus trichocarpa* | NCBI | 14059 | 10473626 | 744.98 | 40.93 | 44.54 ± 17.14 | 18.67 - 84.12 | MseI - CviAII |
| Streptophyta | *Prunus persica* | NCBI | 7062 | 4677442 | 662.34 | 42.50 | 41.86 ± 13.31 | 21.81 - 74.45 | MseI - CviAII |
| Streptophyta | *Solanum lycopersicum* | NCBI | 17849 | 15412230 | 863.48 | 40.55 | 47.90 ± 16.61 | 20.62 - 81.69 | MseI - CviAII |
| Streptophyta | *Solanum tuberosum* | NCBI | 19671 | 15691567 | 797.7 | 40.98 | 48.51 ± 16.99 | 21.89 - 83.79 | MseI - CviAII |
| **Avg. Streptophyta (N=19)** | | | **14288** | **11738143** | **789.03** | **42.32** | **48.83 ± 17.01** | **14.84 - 88.83** |  |
