# Supplementary material for: Eukaryotic transcriptomics in silico: Optimizing cDNA-AFLP efficiency
Source: BMC Genomics. 2009 Nov 30;10:565. doi: 10.1186/1471-2164-10-565 (PMC2797533; doi:10.1186/1471-2164-10-565)
Supplement: Additional file 3 — Duplicate species from ENSEMBL and NCBI databases. Duplicate species from the ENSEMBL and NCBI databases. Average sequence length (Avg Seq Lgt), organismal GC-content (% GC) and the percentage of ambiguous base pairs (% non-ACGT) are indicated. The average pool coverage per enzyme combination, along with maximum and minimum coverage values, are shown. [file 1471-2164-10-565-S3.DOC]

Additional file 3 - Duplicate species from ENSEMBL and NCBI databases

Duplicate species from the Ensembl and Ncbi databases. Average sequence length (Avg Seq Lgt), organismal GC-content (% GC) and the percentage of ambiguous base pairs (% non-ACGT) are indicated. The average pool coverage per enzyme combination, along with maximum and minimum coverage values, are shown.

| **Database** | **ENSEMBL** | | | | | | |  | **NCBI** | | | | | | |
| --- | --- | --- | --- | --- | --- | --- | --- | --- | --- | --- | --- | --- | --- | --- | --- |
| **Species** | **N Seq** | **Total pool size (bp)** | **Avg Seq Lgt** | **% GC** | **% Non-ACGT** | **Coverage ± SD** | **Min-Max Cov.** |  | **N Seq** | **Total pool size (bp)** | **Avg Seq Lgt** | **% GC** | **% Non-ACGT** | **Coverage ± SD** | **Min-Max Cov.** |
| *Aedes aegypti* | 18061 | 27616123 | 1529.1 | 48.0 | 0.02 | 74.9 ± 7.2 | 59.5 - 85.7 |  | 19204 | 25218464 | 1313.2 | 46.4 | 0.07 | 72.1 ± 6.5 | 56.8 - 83.2 |
| *Anopheles gambiae* | 13133 | 20879537 | 1589.9 | 55.3 | 0.19 | 78.1 ± 12.7 | 49.4 - 92.3 |  | 21379 | 15129263 | 707.7 | 52.9 | 0.19 | 64.6 ± 15.8 | 35.3 - 84.3 |
| *Bos taurus* | 28958 | 49808680 | 1720 | 52.5 | 0.01 | 61.6 ± 7.6 | 49.6 - 79.2 |  | 44106 | 61853260 | 1402.4 | 48.9 | 0.02 | 51.5 ± 11.0 | 37.3 - 79.4 |
| *Caenorhabditis elegans* | 28981 | 40353676 | 1392.4 | 42.3 | <0.01 | 63.3 ± 13.2 | 34.7 – 84.0 |  | 21658 | 28571833 | 1319.2 | 41.7 | 0.02 | 62.3 ± 13.8 | 33.7 – 84.0 |
| *Canis familiaris* | 27301 | 42169482 | 1544.6 | 51.9 | <0.01 | 59.5 ± 8.8 | 46.1 - 80.2 |  | 27781 | 39172416 | 1410 | 49.8 | 0.21 | 55.6 ± 9.7 | 42.2 - 78.5 |
| *Ciona intestinalis* | 19858 | 29064597 | 1463.6 | 41.6 | <0.01 | 64.9 ± 13.7 | 39.0 - 90.2 |  | 3494 | 2996310 | 857.6 | 39.5 | 0.28 | 49.6 ± 14.3 | 25.8 - 80.6 |
| *Ciona savignyi* | 20359 | 32691732 | 1605.8 | 45.0 | <0.01 | 71.9 ± 11.0 | 48.1 - 88.7 |  | 7678 | 4396714 | 572.6 | 39.9 | 0.21 | 43.8 ± 14.2 | 20.4 - 75.0 |
| *Danio rerio* | 31841 | 51017126 | 1602.3 | 48.1 | 0.07 | 67.1 ± 8.9 | 50.6 - 84.6 |  | 56561 | 75435852 | 1333.7 | 44.5 | 0.07 | 55.9 ± 10.8 | 40.7 - 86.6 |
| *Drosophila melanogaster* | 20909 | 48315668 | 2310.8 | 49.9 | <0.01 | 84.7 ± 6.2 | 71.8 - 92.3 |  | 17143 | 31940522 | 1863.2 | 48.9 | <0.01 | 76.7 ± 6.6 | 63.9 - 87.3 |
| *Equus caballus* | 27192 | 46568281 | 1712.6 | 50.0 | <0.01 | 56.7 ± 8.0 | 42.8 - 75.3 |  | 8113 | 11844488 | 1459.9 | 48.9 | 0.01 | 56.6 ± 7.9 | 46.1 - 75.5 |
| *Gallus gallus* | 22291 | 39792561 | 1785.1 | 48.6 | <0.01 | 61.4 ± 9.2 | 43.8 - 81.5 |  | 33589 | 53084019 | 1580.4 | 47.3 | 0.38 | 59.0 ± 11.5 | 41.1 - 83.8 |
| *Gasterosteus aculeatus* | 27629 | 45847847 | 1659.4 | 55.0 | <0.01 | 70.7 ± 15.9 | 39.5 - 89.4 |  | 18965 | 26081100 | 1375.2 | 47.4 | 1.26 | 74.4 ± 12.8 | 47.9 - 92.5 |
| *Homo sapiens* | 48803 | 125500000 | 2571.6 | 49.7 | <0.01 | 71.7 ± 6.9 | 62.0 - 85.6 |  | 123808 | 147280000 | 1189.6 | 46.1 | 0.11 | 40.5 ± 13.4 | 24.3 - 76.8 |
| *Macaca mulatta* | 38146 | 70430633 | 1846.3 | 50.4 | 0.01 | 62.2 ± 7.5 | 52.4 - 78.7 |  | 15307 | 40059048 | 2617 | 47.8 | 0.05 | 72.0 ± 7.8 | 59.7 - 87.7 |
| *Monodelphis domestica* | 33279 | 57497869 | 1727.8 | 48.1 | <0.01 | 58.5 ± 11.7 | 38.5 - 83.1 |  | 959 | 1914395 | 1996.2 | 45.5 | <0.01 | 57.4 ± 14.7 | 35.9 - 84.9 |
| *Mus musculus* | 40959 | 99678366 | 2433.6 | 50.0 | <0.01 | 72.0 ± 7.4 | 60.6 – 87.0 |  | 79607 | 113180000 | 1421.8 | 46.7 | 0.17 | 46.3 ± 13.0 | 29.1 - 78.4 |
| *Ornithorhynchus anatinus* | 27383 | 37194655 | 1358.3 | 53.3 | 0.01 | 59.2 ± 10.3 | 40.1 - 79.3 |  | 1688 | 2073163 | 1228.2 | 51.4 | 0.02 | 60.2 ± 9.2 | 41.8 - 77.9 |
| *Oryzias latipes* | 24662 | 38325234 | 1554 | 52.4 | 0.01 | 65.7 ± 12.2 | 44.2 - 82.9 |  | 17373 | 12850714 | 739.7 | 47.6 | 0.21 | 46.8 ± 12.2 | 25.6 - 74.7 |
| *Rattus norvegicus* | 34704 | 60508280 | 1743.6 | 51.1 | 0.01 | 62.6 ± 7.7 | 51.8 - 81.1 |  | 64373 | 70355651 | 1092.9 | 48.2 | 0.18 | 43.6 ± 12.8 | 27.4 - 75.8 |
| *Takifugu rubripes* | 48027 | 91791931 | 1911.3 | 54.0 | <0.01 | 72.1 ± 14.8 | 43.4 - 88.9 |  | 3757 | 2475886 | 659 | 48.9 | 0.02 | 41.4 ± 11.9 | 19.6 - 66.0 |
| *Xenopus tropicalis* | 27711 | 45111427 | 1627.9 | 46.4 | <0.01 | 62.2 ± 9.9 | 47.7 - 84.4 |  | 42522 | 45667114 | 1074 | 42.6 | 0.06 | 46.6 ± 15.5 | 25.1 - 85.0 |
| **Average** | **29057** | **52388748** | **1747** | **49.7** | **<0.01** | **66.7** | **34.7 - 92.3** |  | **29955** | **38646677** | **1296** | **46.7** | **0.17** | **56.0** | **19.6 - 92.5** |
