# Supplementary material for: Eukaryotic transcriptomics in silico: Optimizing cDNA-AFLP efficiency
Source: BMC Genomics. 2009 Nov 30;10:565. doi: 10.1186/1471-2164-10-565 (PMC2797533; doi:10.1186/1471-2164-10-565)
Supplement: Additional file 4 — Influence of database origin on pool coverage. The influence of database origin and enzyme choice on cDNA pool coverage for the 21 species present in both databases. We accounted for variability in coverage resulting from the nesting of species within database and weighted cDNA pool coverage by the number of sequences per pool to account for variation in available sequence data. Denominator degrees of freedom were Kenward-Roger corrected. Partial R-square indicates the proportion of the variation in cDNA pool coverage which is explained by each factor/interaction [24]. [file 1471-2164-10-565-S4.DOC]

**Additional file 4 - Influence of database origin on pool coverage**

The influence of database origin and enzyme choice on cDNA pool coverage for the 21 species present in both databases. We accounted for variability in coverage resulting from the nesting of species within database and weighted cDNA pool coverage by the number of sequences per pool to account for variation in available sequence data. Denominator degrees of freedom were Kenward-Roger corrected. Partial R-square indicates the proportion of the variation in cDNA pool coverage which is explained by each factor/interaction [24].

| **Source** | **Num df** | **Den df** | **F** | **Sig.** | **Partial R-square** |
| --- | --- | --- | --- | --- | --- |
| Model | 106 | 129.31 | 34.44 | <0.001 | 96.58 |
| Database origin | 1 | 17.62 | 0.00 | 0.964 | 0.01 |
| Total pool size (bp) | 1 | 22.66 | 1.49 | 0.234 | 6.18 |
| Average sequence length | 1 | 12.46 | 197.49 | <0.001 | 94.06 |
| GC content | 1 | 14.68 | 9.31 | 0.008 | 38.81 |
| Non-AGCT content | 1 | 14.36 | 26.70 | <0.001 | 65.03 |
| Species | 20 | 11.34 | 21.57 | <0.001 | 97.44 |
| Enzyme combination | 27 | 1055.40 | 61.44 | <.0001 | 61.12 |
| Database origin * Enzyme combination | 27 | 1055.40 | 5.68 | <0.001 | 12.69 |
| Enzyme combination * GC content | 27 | 1055.40 | 9.95 | <0.001 | 20.29 |
